# Improving the accuracy of emergency department clinicians in detecting SARS-COV-2 on chest X-rays using a bespoke virtual training platform

**DOI:** 10.1186/s12909-025-07672-z

**Published:** 2025-08-06

**Authors:** Jasdeep Bahra, Anita Acharya, Sarim Ather, Rachel Benamore, Julie-Ann Moreland, Divyansh Gulati, Lee How, Thandiwe Rosemarysdottir, Miranthi Huwae, Sarah Wilson, Abhishek Banerji, Katerina Manso, Liza Keating, Amy Barrett, Fergus Gleeson, Alex Novak

**Affiliations:** 1 Emergency Medicine Research Oxford (EMROx), Oxford University Hospitals NHS Foundation Trust, Headley Way, Oxford, OX3 9DU UK; 2 Milton Keynes University Hospital, Milton Keynes University Hospital NHS Foundation Trust, Milton Keynes, UK; 3 Wexham Park Hospital, Frimley Health NHS Foundation Trust, Slough, UK; 4 Stoke Mandeville Hospital, Buckinghamshire Healthcare NHS Trust, Aylesbury, UK; 5 Royal Berkshire Hospital, Royal Berkshire NHS Foundation Trust, Reading, UK; 6 Radiology Department, Oxford University Hospitals, Oxford, UK; 7 Pwani University, Kilifi, Kenya

**Keywords:** COVID-19, SARS-CoV-2, Chest X-ray, Radiography, Emergency department, Diagnostic accuracy

## Abstract

**Background:**

During and after the COVID pandemic, online learning became a key component in most undergraduate and post-graduate training. The non-specific symptoms of SARS-CoV-2 and limitations of available diagnostic tests can make it difficult to detect and diagnose in acute care settings. Accurate identification of SARS-CoV-2 related changes on chest x-ray (CXR) by frontline clinicians involved in direct patient care in the Emergency Department (ED) is an important skill. We set out to measure the accuracy of ED clinicians in detecting SARS-CoV-2 changes on CXRs and assess whether this could be improved using an online learning platform.

**Methods:**

Baseline reporting performance of a multi-centre cohort of ED clinicians with varying experience was assessed via the Report and Image Quality Control (RAIQC) online platform. Emergency Medicine clinicians working in EDs across five hospitals in the Thames Valley Emergency medicine Research Network (TaVERN) region were recruited over a six-month period. An image bank was created containing both SARS-CoV-2 and non- SARS-CoV-2 pathological findings. Radiological ground truth diagnosis was established by thoracic radiologists and corroborated by RT- PCR results. Participants then undertook an online training module with performance re-assessed. Diagnostic accuracy and speed of X-ray reporting was assessed before and after training in 3 subgroups: Consultants, Junior Doctors and Nurses.

**Results:**

90 clinicians undertook pre-training assessment and 56 undertook post training assessment. There was an overall improved reporting accuracy for participants who undertook both pre and post training assessments from (44.0±10.5%) to 57.4% (±9.39)% (*p* < 0.001). The sensitivity for recognition of SARS-CoV-2 improved from 64.8 to 76.8%.

**Conclusion:**

ED clinicians show moderate baseline accuracy in the identification of SARS-CoV-2 related changes on CXR. Accuracy and speed can be improved by online training.

## Introduction

The need for social distancing as a direct result of the severe acute respiratory syndrome coronavirus 2 (SARS-CoV-2) pandemic has driven a marked shift towards online learning for healthcare professionals. There has been a steady increase in the utilisation of online learning in medical education which accelerated during the pandemic in 2020. This has been helped by the increase in availability of a variety of learning packages developed to supplement and increase knowledge of both medical students and doctors [1]. There are many available forms of online learning, however interactive tutorials with automatic feedback are a more time-efficient means of learning radiology, compared with peer-reviewed web-based resources [2].

As SARS-CoV-2 transitions from pandemic to endemic, the future prevalence of SARS-CoV-2 is likely to continue to vary. The ability to identify the characteristic radiographic changes in the medical imaging of infected patients will therefore continue to be an important frontline clinical skill, especially for those patients not already clinically suspected of being infected. It will be important for clinicians to be able to accurately discriminate SARS-CoV-2 from other causes of presentations of breathlessness, cough and fever.

The burden of SARS-CoV-2 has proved to be a major challenge within the National Health Service (NHS) and the global population. As of May 2023, there have been 765 million confirmed SARS-CoV-2 cases worldwide, with just over 6.9 million deaths [3]. Of these, the United Kingdom (UK) accounted for 21 million patients with 1 million being admitted to hospital since the start of the pandemic [4].

Distinguishing SARS-CoV-2 from other respiratory and infectious illnesses can be challenging for professionals working on the clinical frontline and requires training and experience. Current secondary care diagnosis of SARS-CoV-2 infection is largely based on clinical suspicion. This is subsequently confirmed by laboratory diagnosis using the standard Reverse Transcription Polymerase Chain Reaction (RT PCR) testing, along with screening for those asymptomatic individuals admitted to hospital for other reasons. Given the limitations associated with PCR in terms of a timely result, secondary care institutions frequently utilise Point-of-Care Testing to generate more rapid provisional assessments of SARS-CoV-2 status. Although these rapid antigen tests (RAT) exhibit good specificity (99%), the sensitivity can vary from 69 to 91% and are also affected by viral loads and time of presentation [5, 6]. RAT testing may not be typically performed without clinical suspicion so diagnosis from imaging becomes important for those asymptomatic patients. The Facilitating AcceLerated Clinical Validation of Novel Diagnostics for SARS-CoV-2 (FALCON-C19) platform study co-ordinated widespread evaluations of the effectiveness of such tests in clinical settings, and outlined their current limitations in terms of acute decision making [7, 8, 9]. Initial diagnosis in the hospital/ Emergency Department (ED) setting is therefore still mostly reliant on the history and examination of the patient coupled with interpretation of routine blood tests and imaging [10].

SARS-CoV-2 invades human host cells by attaching to Angiotensin converting enzyme 2 (ACE2) which has a high expression in lung epithelial cells. This can trigger an inflammatory response which leads to alveolar oedema that manifests as opacities on radiographic imaging [11]. Computed tomography (CT) can provide accurate radiological diagnosis of acute SARS-CoV-2 infection, with quoted sensitivities between 88 and 97% [12]. However, the British Society of Thoracic Imaging (BSTI) has stated that there is only a limited role for CT imaging in the diagnosis of SARS-CoV-2 unless the patient is seriously ill or if PCR is unavailable [13]. In the UK, chest X-Ray (CXR) is the primary imaging modality and one of the key diagnostic tools available to front-line clinicians to diagnose SARS-COV-2 in the acute setting [14].

Typically, CXR images are initially evaluated by frontline clinicians rather than radiologists, and the ability to accurately recognise typical SARS-CoV-2 changes and differentiate them from other respiratory illnesses is critically important in delivering appropriate care for patients. Point of care ultrasound has also been employed as an acute diagnostic imaging tool. However, this requires operators to be in more prolonged close contact with suspected patients in comparison to taking a CXR, placing them at an increased risk of contracting infection. In addition, many EDs will lack an adequate number of portable ultrasound machines and appropriately trained staff to enable widespread use.

The utilisation of Artificial Intelligence (AI) algorithms in the interpretation of SARS-CoV-2 images have also showed some promise in research studies [15, 16]. That said, they are yet to receive regulatory clearance for independent image interpretation and are not integrated with hospital information technology (IT) infrastructure which presents another barrier to their adoption into clinical care [8, 17, 18].

Report and Image Quality Control (RAIQC) is an online simulation platform for medical imaging that enables healthcare workers to enhance their radiology reporting skills [19]. We aimed to measure the accuracy of ED clinicians across the Thames Valley Emergency medicine Research Network (TaVERN– www.tavernresearch.org) in detecting SARS-CoV-2 changes on CXR, and test whether online training could improve the speed and accuracy of frontline clinicians in interpreting CXRs in suspected cases of SARS-CoV-2.

## Methods

### Study design

This diagnostic accuracy study utilised the STARD and STROBE reporting guidelines for overall design and conduct [20, 21]. The study was carried out between May 2020 and February 2021 using the bespoke online training platform Report and Image Quality Control (RAIQC), developed at Oxford University Hospitals (www.raiqc.com).

The platform is accessible through a web-browser from anywhere in the world and combines a zero-footprint web based Digital Imaging and Communications in Medicine (DICOM) viewer with structured reporting templates to deliver teaching, training, and assessment material. For the purposes of the study, a bespoke reporting template was created based on the CXR reporting proforma for suspected SARS-CoV-2 patients issued by the BSTI [22]. This divides patients into four distinct categories based on plain radiographic appearance:

- Normal: No radiographic abnormality detected.
- Classic/Probable SARS-CoV-2: Lower lobe and peripheral predominant multiple opacities that are bilateral.
- Non-SARS-CoV-2: Pneumothorax / non-SARS-CoV-2 infection / Pleural effusion/ Pulmonary oedema.
- Indeterminate of SARS-CoV-2: Does not fit Classic or Non-SARS-CoV-2 descriptors.

A teaching slideshow was created by two experienced thoracic radiologists at Oxford University Hospitals with examples of the imaging features for each category. Assessment and training modules were created using two unique sets of 30 images each (7 normal, 7 SARS-CoV-2, 6 indeterminate, 4 non-SARS-CoV-2 infection, 2 pneumothorax, 2 pleural effusion and 2 pulmonary oedema). Chest X-rays were initially identified by searching the radiology information system at Oxford University Hospitals. The images were then reviewed by two thoracic radiologists. Disagreement between the reviewers on the classification of radiographs (*n* = 3) was resolved through consensus before finalising the content of the module. In addition to this, the RT-PCR results were reviewed for each case to ensure that all the SARS-CoV-2 cases were RT-PCR positive while all the non-SARS-CoV-2 19 cases were RT-PCR negative.

When reviewing the online images, a short vignette with the presenting complaint was provided for readers for each image. They were then required to review the CXR image itself, choose a diagnostic category and click on the abnormality location before submitting the case. All readers could complete this process from remote locations should they wish, so long as an internet connection was present. The readers were asked to complete a pre-training assessment where they were blinded to the ground truth reports.

**Fig. 1:**
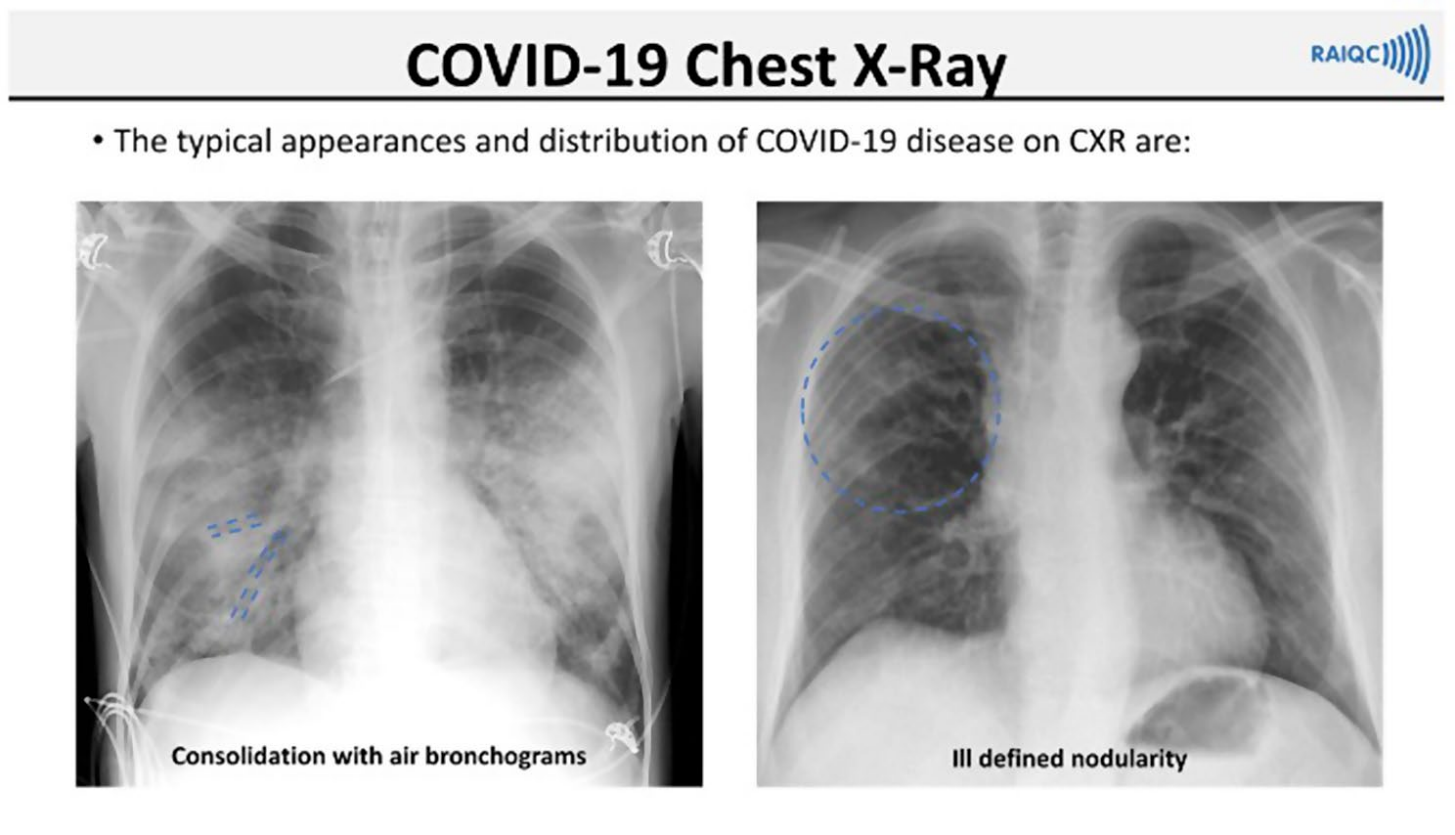
Teaching slide showing typical appearance of SARS-CoV-2 infection on chest X-ray

**Fig. 2:**
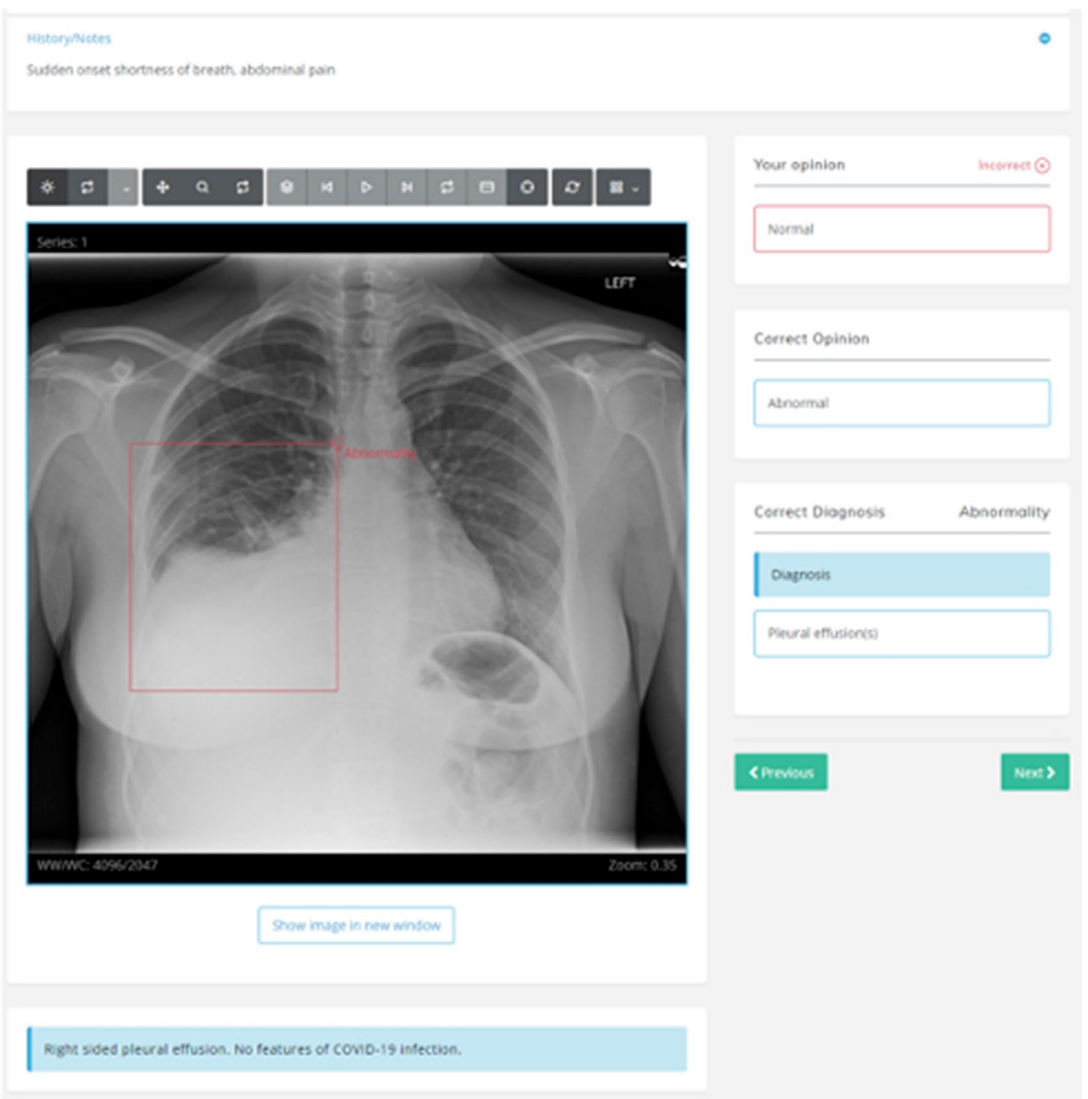
A training case with the abnormality outlined and correct diagnosis provided

Following this they were given access to the teaching slideshow (27 slides) and the training set of cases for self-directed learning. [Figures 1 and 2] After each case they were informed of the correct diagnosis, along with additional teaching notes. Upon completion of the training module, the readers completed another blinded assessment module, based on the same cases as the pre-training assessment. During both modules participants were instructed to complete the entire test with technical issues addressed but no discussion or clarification was had about test images to avoid bias. No personalised feedback was given until the study closed.

### Participants

The study population included consultants, junior doctors and nurses working in EDs from across the Thames Valley Emergency Medicine Research Network. Nurses of all grades were invited to represent their increasing role in interpretation of clinical results (including radiographs) prior to review by the treating emergency clinician, with the perceived benefit of earlier identification of concerning pathologies. Participants were recruited through advertising during departmental handovers, with posters and other electronic staff communications. Statistical analysis was performed on all participants who undertook the baseline and final assessments.

### Testing methods

Data was collected on the time taken for each clinician to review individual cases via the RAIQC platform which timestamps when a case is first opened and when it is submitted. The number of overall cases answered correctly and breakdown for individual pathologies was also recorded. The average scores for the pre- and post-training assessments were compared to assess for improvements to diagnostic accuracy and speed.

### Analysis

We expected to find improvement in both accuracy and speed. The calculated sample size was 97 pairs using a power calculator for a paired t-test with an alpha of 0.05, power 0.9, and an estimated accuracy increase of 10% and SD of 30%. Statistical analysis was carried out using Microsoft Excel version 16.63.1. The reporting accuracy mean, standard deviation, median, 25th and 75th quartiles were calculated for both baseline and post-training assessment modules. The mean and standard deviation of time taken to evaluate each case was also calculated. Paired t-tests were carried out to assess difference in pre and post training assessments. In addition, the case submissions were pooled to compare the change in reporting accuracy between groups and for pathology sub types.

A subset of participants undertook the modules on more than one occasion. In these cases, only the first attempt was used in the statistical analysis. Participants were also excluded from the statistical analysis if they had attempted less than 90% of cases in the assessment module.

### Ethics and public engagement

As per Health Research Agency guidance (https://www.hra-decisiontools.org.uk/research/), this study constitutes Observational Research and did not require ethical committee review. Clinical data was used under existing data governance framework between RAIQC Ltd and Oxford University Hospitals NHS Foundation Trust. The collection and aggregation of the retrospective dataset was undertaken under an approved data protection impact assessment undertaken by Oxford Hospitals NHS Foundation Trust. It was also discussed at our local ACUTE Care Patient and Public Involvement group to facilitate discussion of the study and help direct its design, development and implementation. The study development team included a Consultant and Senior Registrar from the Emergency Department who advised on the design and implementation of the study. Written consent was provided by participants for use of anonymised and aggregated data.

### Outcomes

Our primary outcome was to evaluate the change in accuracy of ED clinicians’ interpretation by using an online learning platform. Our secondary outcome was to observe any change in time taken to report images.

## Results

**Fig. 3:**
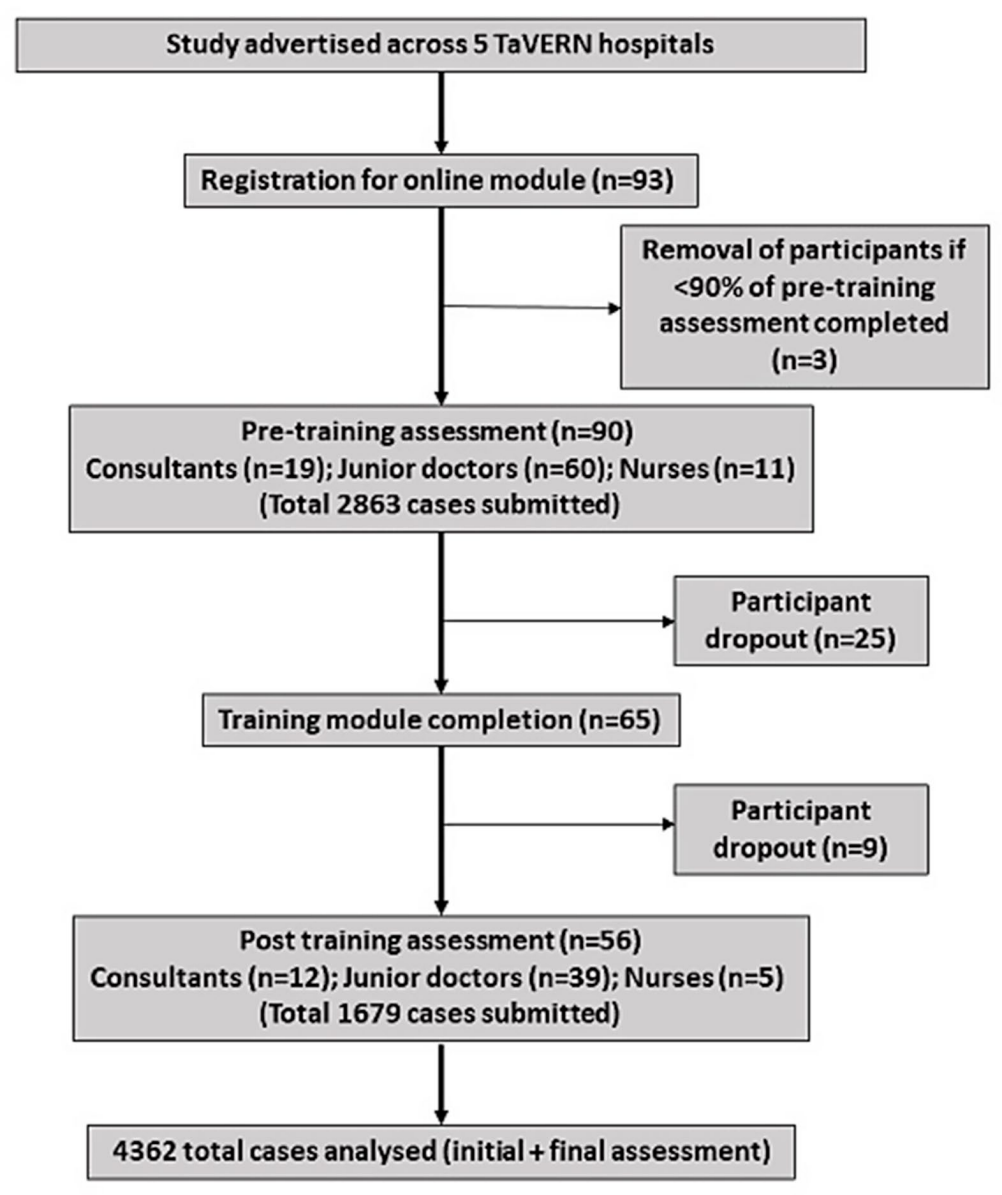
Study flow diagram

### Reader participation and performance

**Fig. 4:**
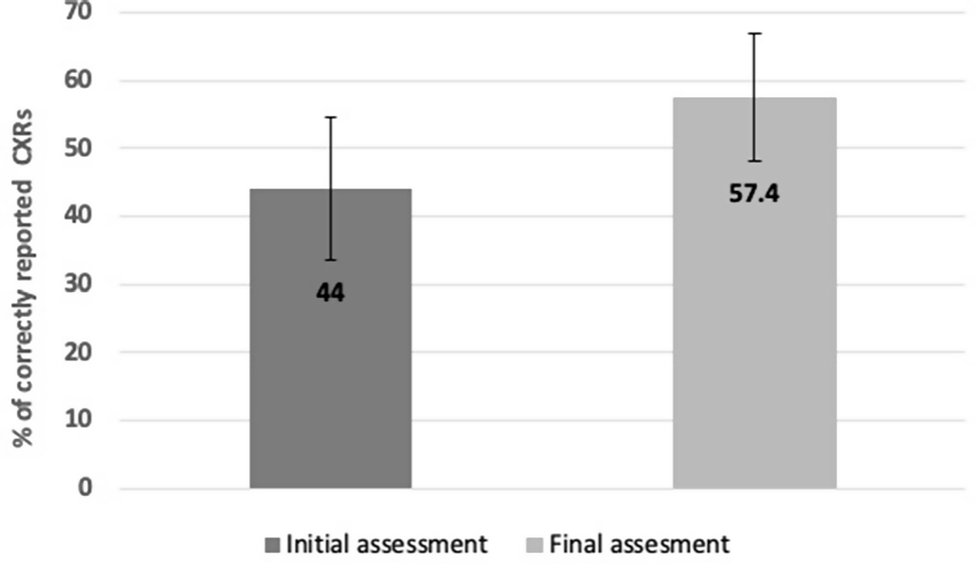
Mean accuracy for participants completing full assessment (*n* = 56)

A flowchart summarising the progress of the study is presented in Fig. 3, and a breakdown of participants is summarised in Table 1. [Fig. 3; Table 1] 90 clinicians undertook the pre-training assessment with an overall mean reporting accuracy of 43.8 (±9.89)% across all cases. There was no statistically significant difference (*p* = 0.670) in the baseline diagnostic accuracy of the 34 individuals who dropped out of the study after completing the initial assessment (43.1±9.01%) compared with the 56 participants who completed the pre- and post-training assessment (44.0±10.5%). For the 56 participants who completed the post-training assessment, this reporting accuracy improved to 57.4 (±9.39%) (*p* < 0.001). [Fig. 4] Mean time for completion of the training cases was 35 (± 12.5) mins. Mean time taken for training slides was not recorded. Of the total healthcare professionals completing the initial module, junior doctors comprised 67% (*n* = 60), followed by consultants at 21% (*n* = 19) and then nurses at 12% (*n* = 11). From the readers undertaking the initial assessment 63% of consultants (*n* = 12), 65% of junior doctors (*n* = 39) and 45% of nurses (*n* = 5) went on to complete the final assessment. After completion of the e-learning module, overall reporting time per case for all staff groups reduced from 57 to 53 s but this change was not statistically significant (*p* = 0.269).

**Table 1:** Distribution and seniority/role of participants completing initial and final assessments

	Initial Assessment					Final Assessment		
	**Total**	**Junior Doctor**	**Consultant**	**Nurse**	**Total**	**Junior Doctor**	**Consultant**	**Nurse**
Oxford University Hospitals	**37**	16	12	9	**21**	8	9	4
Wexham Park Hospital	**28**	23	5	0	**16**	14	2	0
Royal Berkshire Hospital	**17**	15	1	1	**14**	12	1	1
Stoke Mandeville Hospital	**7**	5	1	1	**4**	4	0	0
Milton Keynes Hospital	**1**	1	0	0	**1**	1	0	0
Total	**90**	**60**	**19**	**11**	**56**	**39**	**12**	**5**

### Pooled analysis of accuracy

**Fig. 5:**
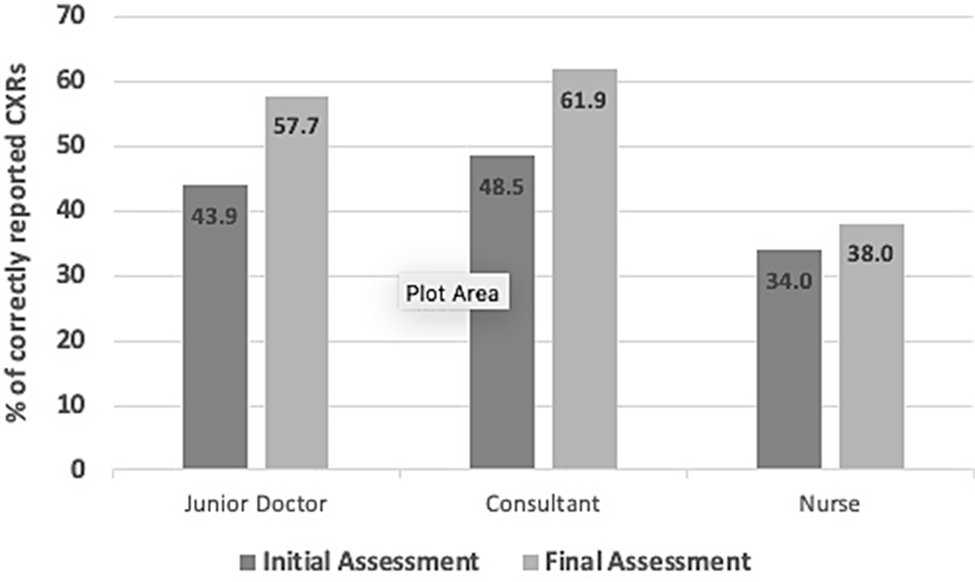
Mean pooled sub-group accuracy of CXR reporting in all pathologies by role

**Fig. 6:**
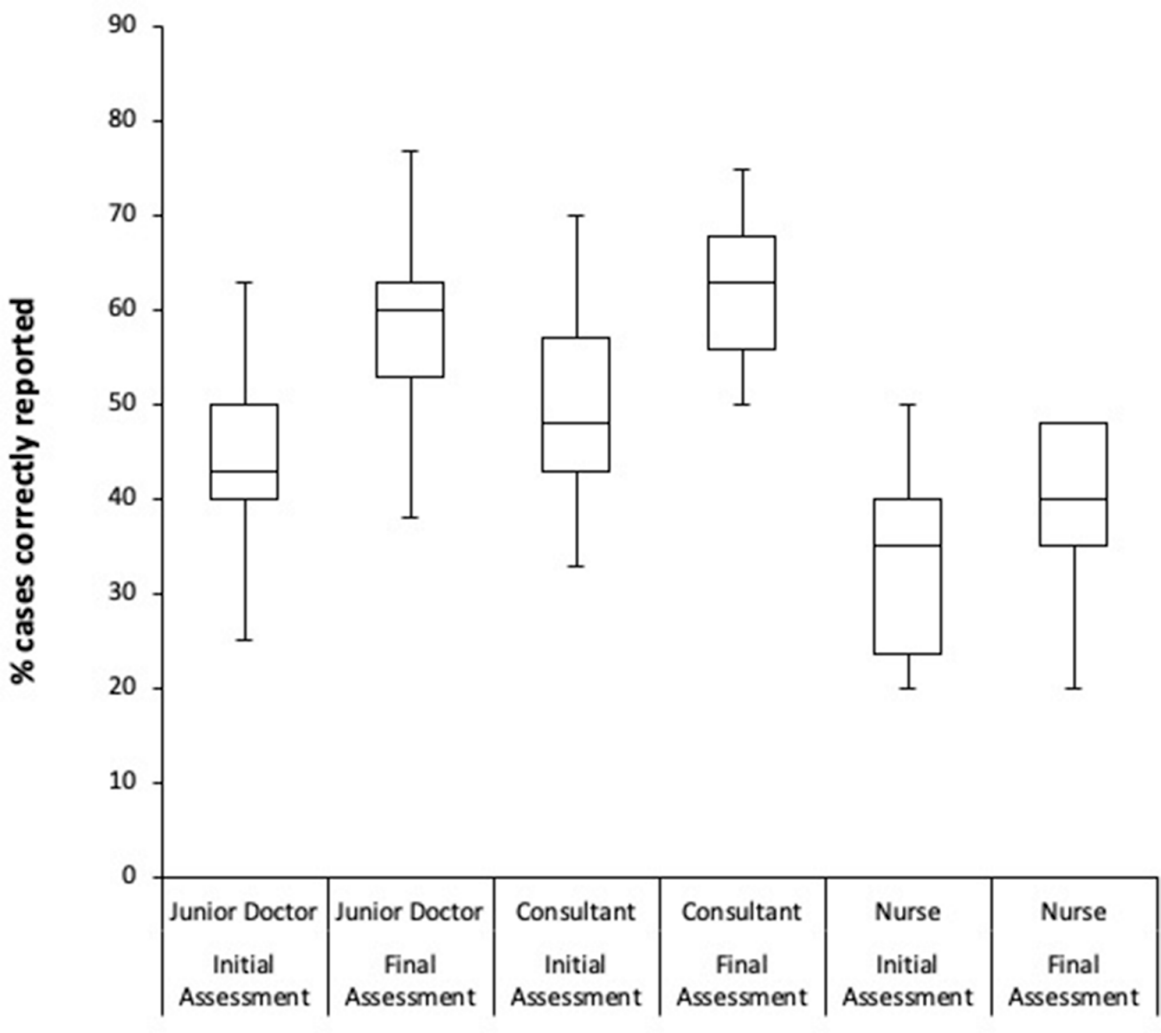
Median accuracy of reader subgroups in CXR Reporting– participants in initial and final assessments

Overall, 90 readers submitted 2683 cases as part of the initial online assessment. Of these, 56 went on to complete the training and final assessment. 1679 cases were submitted as part of the final assessment. Overall, the readers correctly interpreted 1172 out of the 2683 submitted cases in the initial assessment and 954 out 1679 cases in the final assessment. Pooled analysis of all submitted cases demonstrated an overall increase in the % of correctly reported cases from 43.7 to 56.8%. The junior doctor group demonstrated the greatest improvement (Mean 43.9–57.7%, Median 43–60%), followed by the consultants (mean 48.5–61.9%, median 48–63%) and nurses (mean 34–38%, median 35–40%). [Table 2; Figs. 5 and 6]

**Table 2:** Median accuracies of clinical subgroups across initial and final assessments as percentages

	Initial Assessment	Final Assessment	Initial Assessment	Final Assessment	Initial Assessment	Final Assessment
Role	Junior Doctor	Junior Doctor	Consultant	Consultant	Nurse	Nurse
Min	23	37	33	50	20	20
Q_1_	40	53	43	55.75	23.5	35
Median	43	60	48	63	35	40
Q_3_	50	63	57	67.75	40	48
Max	63	77	70	75	50	48
IQR	10	10	14	12	16.5	13

**Fig. 7:**
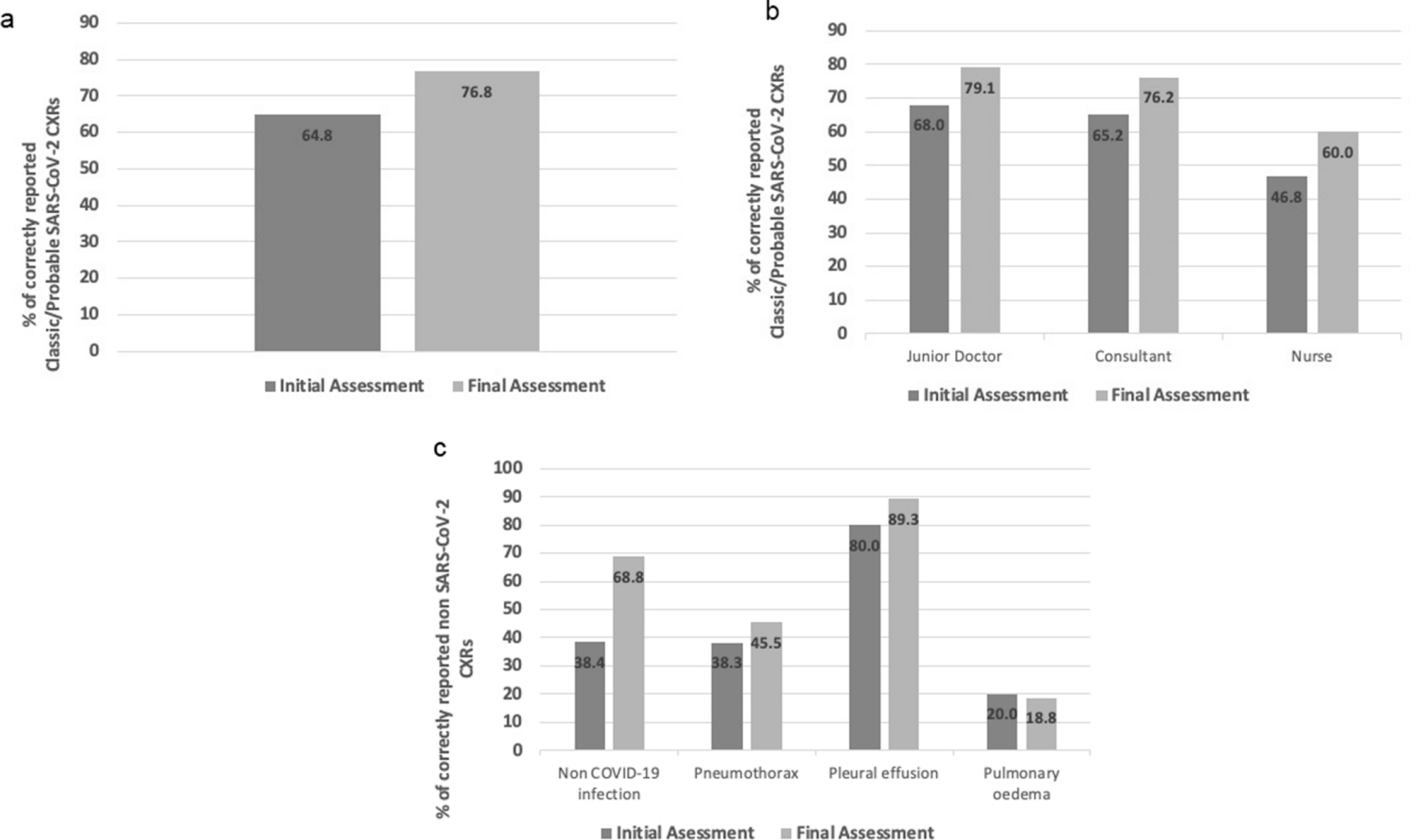
**a**: Pooled accuracy of Classic/Probable SARS-CoV-2 CXR Reporting– participants IA (90), FA (56). **b**. Subgroup accuracy of pooled CXR reporting in COVID-19. **c**. Pooled accuracy of CXR reporting in non-COVID-19 cases

Pooled accuracy was higher when reporting only the radiographical presence/absence of SARS-CoV-2 changes, with an overall pooled accuracy of 64.8% across all groups in the initial assessment. A rise in accuracy (12%) of reporting was seen in all SARS-CoV-2 cases from 64.8 to 76.8% [Fig. 7a] which was reflected across all sub-groups. [Fig. 7b] Accuracy for diagnosing non-SARS-CoV-2 infection cases improved from 38.4 to 68.8% across all reporters. [Fig. 7c] The full breakdown of results across all pathologies is shown below. [Table 3]

**Table 3:** Pooled cases across all reader groups with percentage of correct diagnosis in all pathologies

Pathology	Submitted initial cases	% Correct (*n*)	Submitted final cases	% Correct (*n*)
Classic/Probable COVID-19	628	64.8 (407)	392	76.8 (301)
Indeterminate/poor quality radiograph	543	26.2 (142)	336	48.5 (163)
Other Non-SARS-COV-2 abnormality:	886	43.1 (382)	560	58.2 (326)
- Non COVID-19 infection	346	38.4 (133)	224	68.8 (154)
- Pneumothorax	180	38.3 (69)	112	45.5 (51)
- Pleural effusion(s)	180	80.0 (144)	112	89.3 (100)
- Pulmonary oedema	180	20.0 (36)	112	18.8 (21)
Normal	629	38.5 (242)	391	41.9 (164)
Abnormal	2054	45.3 (930)	1288	61.3 (790)
Overall	2683	43.7 (1172)	1679	56.8 (954)

## Discussion

We undertook a study evaluating the impact of online training on the diagnostic accuracy of ED clinicians when identifying characteristic SARS-CoV-2 changes on CXRs. Our findings, indicate that 56 clinicians (62%) completed the full training and assessments package, resulting in a notable improvement in diagnostic performance. Specifically, chest X-ray interpretation accuracy increased by 13.4% (from 44 to 57.4%) (*p* < 0.001), while pooled analysis of accuracy in reporting SARS-CoV-2 cases rose by 12% (from 64.8 to 76.8%). This suggests that the training intervention had a measurable impact on clinician performance in these key diagnostic areas. There was no significant difference in the speed of image interpretation.

Literature is scant regarding specific evaluation of diagnostic accuracy and speed amongst ED clinicians using e-learning packages. Ogura et al. showed similar results with an overall increase in accuracy in medical students (34.5–72.7%) when using an online CXR learning package [23]. A study evaluating e-learning in CT scans was performed by Groth et al. which showed significantly better performance for detecting specific pathologies, further supporting the increase in accuracy shown in our results [24]. Dropout rates were not specifically commented on in these studies, and both were smaller single-centre studies consisting of 10 and 38 participants respectively, which limits direct comparison.

Initial CXR is a useful imaging modality in the diagnosis of SARS-CoV-2, with a sensitivity and specificity quoted at 61% and 76% when reported by senior radiologists [25]. A recent Cochrane review highlighted the diagnostic accuracy of CXR in detection of SARS-CoV-2. From 17 studies the pooled sensitivity of chest X-ray was 73.1% (95% CI 64.1 to 80.5), and pooled specificity was 73.3% (95% CI 61.9 to 82.2) [26]. Improvements in accuracy with ED clinician experience suggests training of junior ED clinicians in the interpretation of Sars-CoV-2 related CXR changes might be beneficial, further supporting the use of online educational tools [27]. Junior doctors may have shown greater improvement than consultants in online CXR learning during the early pandemic because they started with lower baseline experience and were more adaptable to new diagnostic patterns. Their comfort with digital tools and strong motivation to learn likely enhanced engagement and uptake [28]. In contrast, consultants may have relied on established heuristics, limiting the degree of change. Nurses also exhibit a spectrum of ability with interpreting CXRs especially with the advent of advanced nurse practitioners. This platform would also lend to the training of all nurses, with a range of abilities, many of whom are increasingly involved in the primary interpretation of CXRs. *Murphy et al.* showed that artificial intelligence systems may also play a role in increasing accuracy of Sars-CoV-2 diagnosis with CXRs, with detection comparable to that of six independent readers [18].

We measured the reporting accuracy and speed of 90 Emergency Medicine clinicians across the Thames Valley Emergency Medicine Research Network in correctly identifying typical SARS-CoV-2 changes on CXRs. Initial pooled accuracy for the detection of SARS-CoV-2 changes on CXR was found to be 64.8% across all reader groups, increasing to 76.8% after completion of the e-learning module.

The results showed a disproportionate increase in accuracy of infection compared with non-infectious pathologies. This is most probably due to the teaching slides and training cases primarily being focused on CXR findings of SARS-CoV-2 and its differentiation from other infections. Detection of pulmonary oedema was poor amongst all groups. This could be because like SARS-COV-2, acute pulmonary oedema also presents with bilateral airspace opacification, and it is therefore difficult to differentiate between the two conditions on imaging alone. Cases in the indeterminate category were also poorly identified by all participating groups. A recent study validating the BSTI guidelines, upon which the reporting template was based, found only slight interobserver agreement amongst consultant thoracic radiologists. BSTI suggested that this category should be combined with “non-SARS-CoV-2” as there is an inherent overlap of CXR appearances between these. Examples of this exist in patients with limited or unilateral consolidation, which could be SARS-CoV-2 or bacterial in aetiology; and in patients with multiple radiological abnormalities, for example, fluid overload and alveolar opacity [29].

### Strengths

This multicentred study was conducted using ED clinicians from 5 different TaVERN hospital sites. These included both teaching and district general hospitals with ED clinicians of varying role and grade in an attempt to broaden the participant group as much as possible and increase the generalisability of the findings.

Mixing SARS-CoV-2 CXR images with those containing other pathologies in the image bank increased the challenge to the clinicians and provided a closer comparison to a real-world setting than comparing against normal CXR images alone. The measurement of time taken to report as well as diagnostic accuracy allowed an assessment of the potential impact of introducing widespread online training on clinicians’ efficiency as well as accuracy.

The online training learning process was relatively quick, with a mean time to completion of training cases of 35 (± 12.5) mins, though we did not measure time taken to review the 27 training slides which preceded the cases. This ability for remote login allowed participants to compete it in a flexible timeframe in a home setting if desired offering the potential for rapid expansion of the training programme if required.

The approach to self-guided improvement of front-line staff CXR interpretation has potentially much wider applications than just SARS-CoV-2, particularly in the accurate detection of other acute pathologies which significantly improved in pooled analysis. This could also be expanded to include other imaging modalities, and could be easily and rapidly scalable to wider healthcare networks.

### Limitations

This multicentre study was conducted within a single UK region, potentially limiting generalizability despite its diverse hospital settings and clinician workforce.

Only 62% of participants completed the full training, below the target sample size of 97 participants. Nurses had the highest dropout rate, though their small numbers likely did not affect overall findings. This might reflect the current perception of nurses that they do not need this skill in their current role. Furthermore, there could have been concerns regarding the time to taken to complete the package given it would not add to their practice. Despite this high dropout rate, 4362 images were reviewed as part of this study, yielding a large dataset with a clear increase in performance between the two assessments, meaning the results can still be regarded as significant.

Another limitation is the relatively small number of cases within each non-SARS-CoV-2 pathology category. Additionally, around 5% of these cases required consensus due to initial disagreement between specialist radiologists. While consensus was reached, this reflects the interpretive complexity of some cases and may illustrate the challenges faced by clinicians without specialist expertise. This has important implications for education and training, highlighting the need to prepare non-specialist clinicians for the diagnostic uncertainty they may encounter in real-world settings.

Post-training surveys were not conducted, making it difficult to determine reasons for dropout. Possible factors include pandemic-related clinical pressures, fatigue, and training duration exceeding expectations. However, the observed performance improvement suggests the package was beneficial for those who completed it.

We did not record the time interval between training completion and the second assessment, which may have varied between participants. As delay between education and post-testing can influence diagnostic accuracy, this represents a limitation of our study and should be addressed in future research.

The study occurred early in the pandemic, when SARS-CoV-2 knowledge was evolving, which may have introduced bias. However, the short study duration likely minimized this effect. Selection bias may have occurred as participation was voluntary, favouring motivated individuals. Future studies should consider broader recruitment and incentives.

Clinicians had varying pre-study experience, particularly nurses who do not routinely interpret CXRs. Their inclusion reflected the changing scope of practice for nurses, whilst also assessing training impact on those with minimal prior exposure and reflecting their expanding role in EDs. The exclusion of other CXR-interpreting specialties (e.g., Acute Medicine, Intensive Care Unit (ICU), Radiographers) limits generalizability, warranting their inclusion in future studies.

Real-world application and long-term skill retention were not assessed due to budget and scope constraints. While immediate post-intervention performance provides valuable insight into short-term learning gains, it does not capture the durability of learning over time. Future studies incorporating delayed post-tests would help evaluate the long-term impact of the training and its relevance to clinical practice.

## Conclusions

In our study ED clinicians demonstrated a moderate baseline accuracy in detecting SARS-CoV-2 on CXR images compared to radiologists and PCR test results [30]. This diagnostic accuracy can be improved by using online tutorials, with the potential for this approach to apply to other pathologies and imaging modalities.

## Data Availability

The datasets used and/or analysed during the current study are available from the corresponding author on reasonable request.

## Declarations

### Study design

As per STROBE guidelines for Observational studies, all methods were carried out in accordance with these guidelines and regulations.

## Abbreviations

ACE2: Angiotensin-converting enzyme 2
BSTI: British Society of Thoracic Imaging
CT: Computed tomography
CXR: Chest X-ray
DICOM: Digital Imaging and Communications in Medicine
ED: Emergency department
FALCONC19: Facilitating AcceLerated Clinical Validation of Novel Diagnostics for SARS-CoV-2
IQR: Interquartile range
NHS: National Health Service
PCR / RTPCR: Polymerase chain reaction / Reverse transcription PCR
RAIQC: Report and Image Quality Control
RAT: Rapid antigen test
SARS-CoV-2: Severe acute respiratory syndrome coronavirus 2
STARD: Standards for Reporting of Diagnostic Accuracy Studies
STROBE: Strengthening the Reporting of Observational Studies in Epidemiology
TaVERN: Thames Valley Emergency Medicine Research Network
UK: United Kingdom
